# Prevention of prescription opioid use disorder

**DOI:** 10.1192/j.eurpsy.2025.94

**Published:** 2025-08-26

**Authors:** F. Fonseca, RD24/0003/0001 co-funded by Instituto de Salud Carlos III (ISCIII) and by the European Union

**Affiliations:** 1 Mental Health Institute, Hospital del Mar Research Institute; 2 Medicine and Life Sciences Department, Universitat Pompeu Fabra, Barcelona, Spain

## Abstract

**Abstract:**

It is important to address the prevention and early detection of opioid addiction with a comprehensive approach. The collaboration and early attention are essential to mitigate the risks associated with opioid misuse. In this session we will review the myths associated with high risk of opioid addiction and how to address when it has been developed.

Some key strategies could be: Provide accurate and understandable information about the risks associated with opioid use, as well as the early signs of addiction. Consider alternative options for pain management, such as physical therapies, exercise, and medications non-opioids, before prescribing opioids. It is important balance pain relief with control of possible addiction risks. The appropriate and controlled indication of opioids is of vital importance to prevent and detect inappropriate use. Prescribe the lowest dose and duration shortest possible. It is important to visit frequent monitoring to detect signs of misuse and/or addiction.

**Disclosure of Interest:**

None Declared

